# Supplementary material for: Host-guest charge transfer for scalable single crystal epitaxy of a metal-organic framework
Source: Commun Mater. 2024 Oct 9;5(1):220. doi: 10.1038/s43246-024-00657-3 (PMC11488492; doi:10.1038/s43246-024-00657-3)
Supplement: Supplementary file 2 — Supplementary Information [file 43246_2024_657_MOESM2_ESM.pdf]

Supplementary Information

Host-guest charge transfer for scalable single crystal epitaxy of a metal-organic framework

Arthur Mantel,[1] **Berthold Stöger,**[2] **Alexander Prado-Roller,**[3] **and Hidetsugu Shiozawa**[1,4*]

[1]J. Heyrovsky Institute of Physical Chemistry, Czech Academy of Sciences, Dolejskova 3, 182 23 Prague 8, Czech Republic

[2]X-ray Centre, TU Wien, Getreidemarkt 9, 1060 Vienna, Austria

[3]Department of Inorganic Chemistry, University of Vienna, Währinger Straße 42, 1090 Vienna, Austria

[4]Faculty of Physics, University of Vienna, Boltzmanngasse 5, 1090 Vienna, Austria

*To whom correspondence should be addressed; E-mail: hide.shiozawa@jh-inst.cas.cz & hidetsugu.shiozawa@univie.ac.at

S1 Structure

S1.1 X-ray diffraction

Crystals were cut with a knife and fragments of suitable size were selected under a polarizing microscope. Intensity data were collected using Cu$K\alpha$ radiation on a STOE STADIVARI diffractometer system equipped with an DECTRIS EIGER CdTe detector. Data were reduced to intensity values using X-Area and a correction for absorption effects applied using LANA [1]. Figure S1a shows the diffraction pattern on the hk0 plane for a fragment for which the crystal structure was determined. The diffraction spots correspond to a $\sim$ b = 21.8 Å. Although the lattice of the crystal was clearly tetragonal, one dimensional diffuse scattering in [100] and [010] direction observed on the hk0 plane suggested a lower symmetry combined with twinning. In fact, no reasonable structure solution was obtained using SHELXT [2] in the either the $4/m$ or the $4/mmm$ Laue groups. Structure solution was successful in the monoclinic crystal system (unique axis [001] of the tetragonal cell). After a few initial refinement cycles with SHELXL [3], application of the ADDSYM routine of PLATON [4] suggested the orthorhombic space group *Pbca* as the most likely symmetry. The missing symmetry of the tetragonal lattice was implemented as twin operation. Since the methyl groups of the DMSO molecules and the disordered solvent molecules could not be properly refined, they were omitted from the final model and their electron density removed using the SQUEEZE routine of PLATON [4].

Figure S1b shows the micrograph of an as-grown microcrystal aligned on the sample holder from which the crystallographic orientations were identified.

(a) (b)

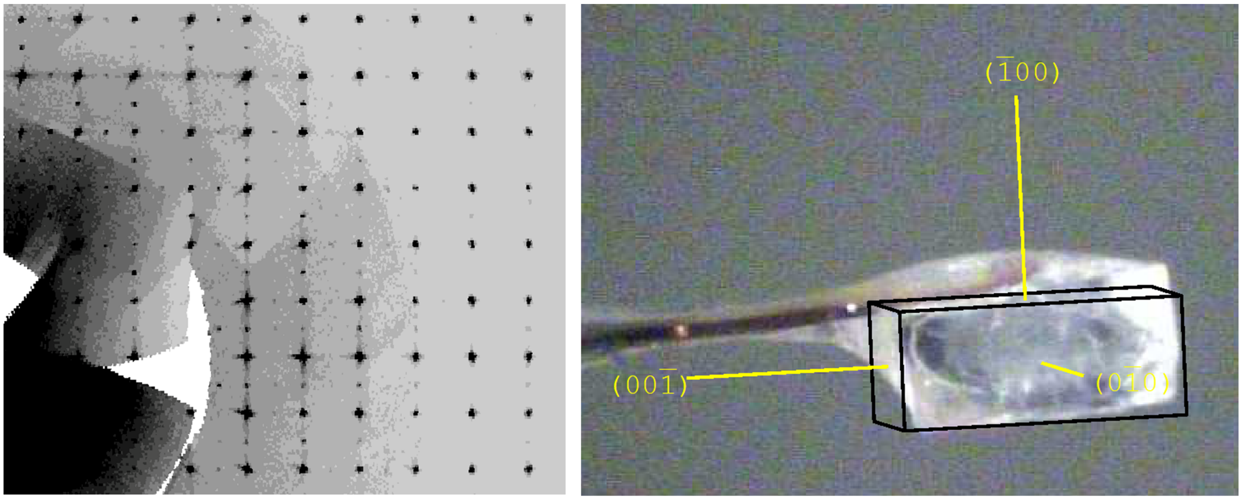

Figure S1: (a) Diffraction pattern on the hk0 plane of a crystal for which the crystal structure was determined. (b) Micrograph of an as-grown microcrystal aligned on the sample holder in order to identify the crystallographic orientations. The crystal dimensions are $0.12 \times 0.08 \times 0.04$ mm. The (100), (010) and (001) faces are perpendicular to axes a, b and c, respectively.

S1.2 X-ray diffraction

Figure S2 display the the structural surfaces visualized with a resolution of 0.2 Åusing Olex2, calcvoid [5].

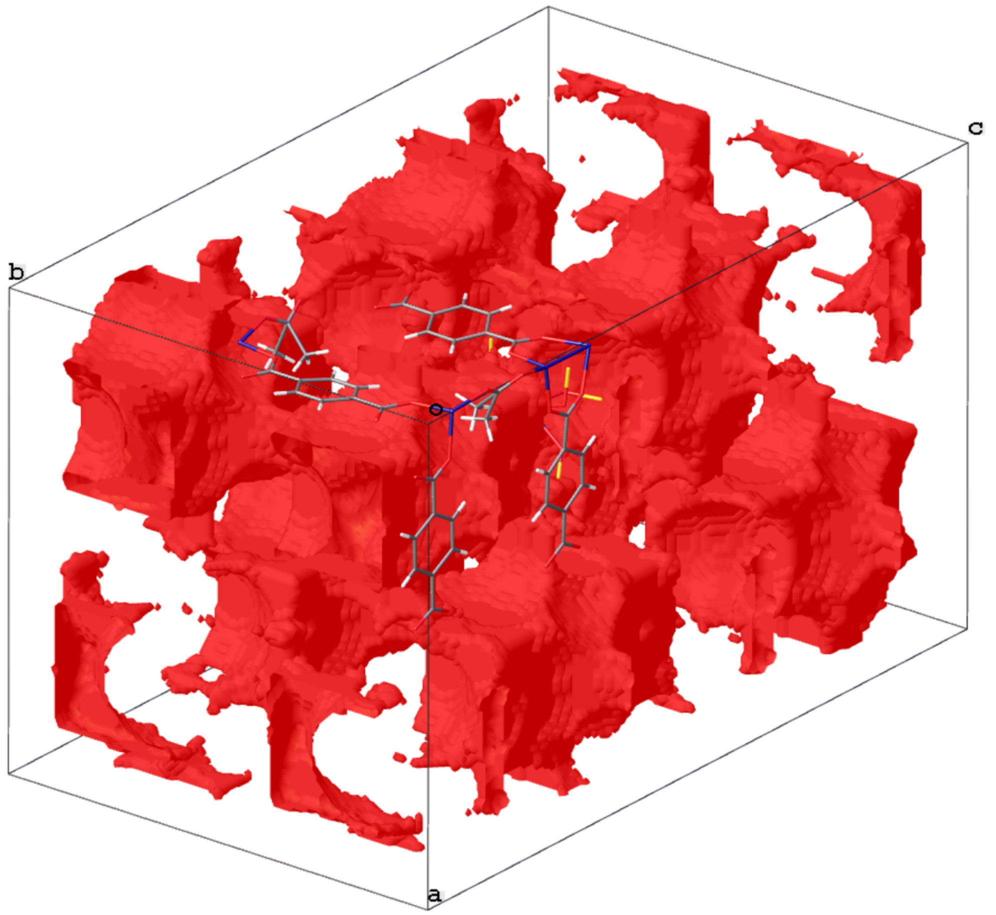

Figure S2: The structural surfaces visualized with a resolution of 0.2 Å.

S1.3 DFT-optimized structure

Figure S3 shows the DFT-optimized crystal structure of the MOF encapsulating the $Zn(DMSO)_6{}^{2+}$ molecules, viewed along the a axis (a), the b axis (b) and the c axis (c), and an arbitrary direction (d). The structure of the $Zn(DMSO)_6{}^{2+}$ molecule in one orthorhombic void has been optimized by DFT calculations, and the structures of the other seven molecules have been made by respective operations of inversion, mirror or rotational symmetry. In panels a, b, and c, the $Zn(DMSO)_6{}^{2+}$ molecules in the front orthorhombic voids are multi-colored, while the other four in the voids behind the first voids are colored in green. In panel d, the $Zn(DMSO)_6{}^{2+}$ molecules in the top orthorhombic voids are multi-colored, while the other four in the bottom voids are colored in green.

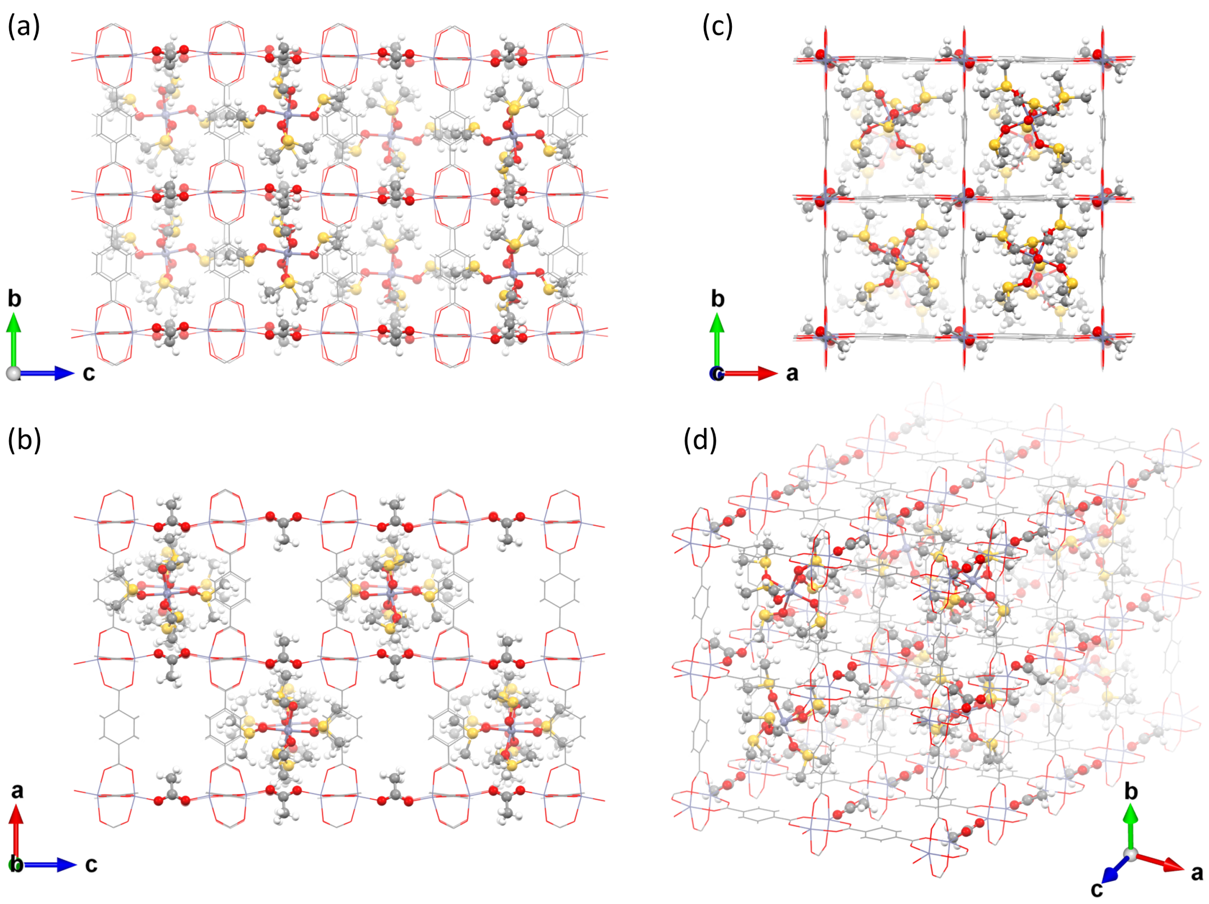

Figure S3: Crystal structure of the Zn-TPA MOF encapsulating the $Zn(DMSO)_6{}^{2+}$ molecules, viewed along the a axis (a), the b axis (b) and the c axis (c), and an arbitrary direction (d). The structure of the $Zn(DMSO)_6{}^{2+}$ molecule has been optimized by DFT calculations.

S2 Homoepitaxy over months

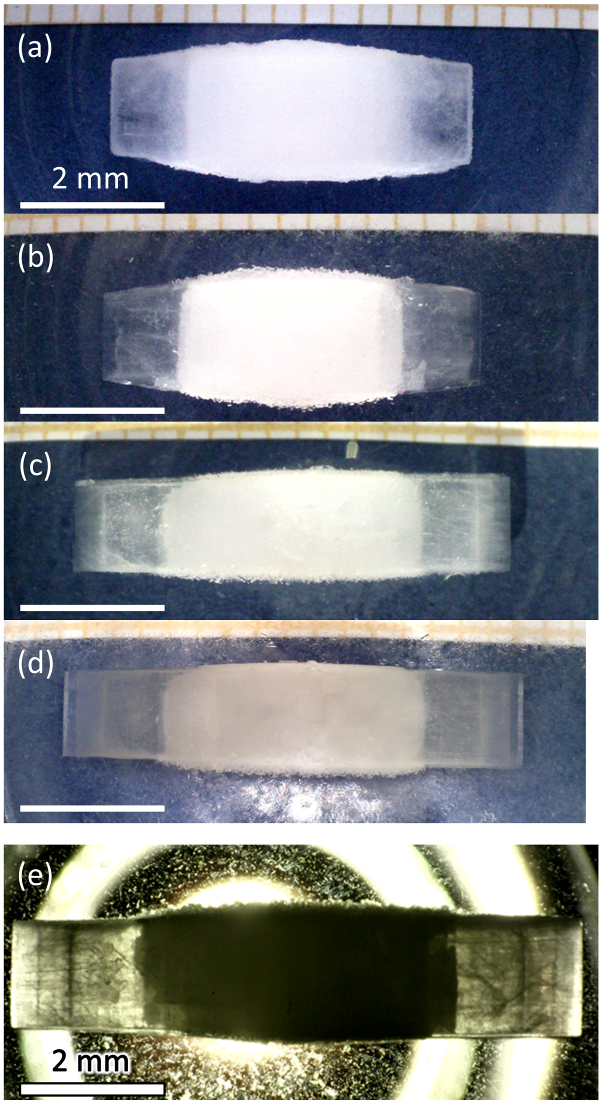

Figure S4: Photographs of the crystal taken after (a) 89 days (b) 109 days (c) 121 days (d) 134 days and (e) 146 days of the multiple reaction steps. The crystal in panel (e) is back-illuminated. Its dimensions reach 19.8 × 4.2 × 4.2 mm.

S3 Stability

The stability of MOF crystals was tested in different environments.

Figure S5 shows a crystal before (A) and after (B) being soaked in the mother solution (the molar ratio of ZnOAc to TPA is 3.0 to 1.0) for 40 minutes in a hermetically sealed cuvette. No changes can be noted.

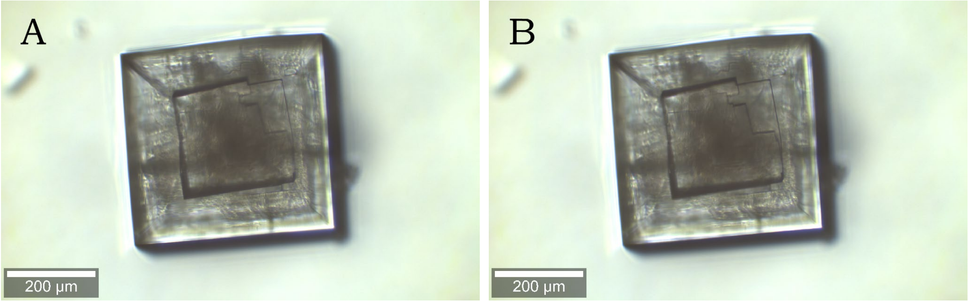

Figure S5: MOF-5 before (A) and after (B) 40 min in the mother solution hermetically sealed in a cuvette.

Figure S6 shows a crystal before (A) and after (B) being soaked in the mother solution (the molar ratio of ZnOAc to TPA is 3.0 to 1.0) for 40 minutes. The solution was exposed to air.

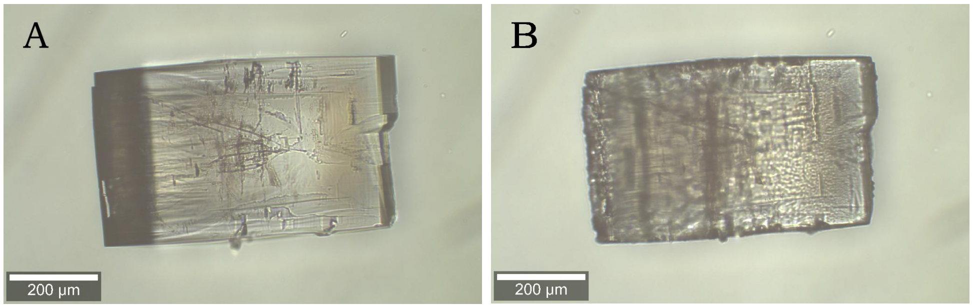

Figure S6: MOF-5 before (A) and after (B) 40 min in the mother solution.

Figure S7 shows a crystal before (A) and after (B) being soaked in DMSO for 40 minutes. The solution was exposed to air. There are no significant changes, but small particles on the surface of the crystal can be noted.

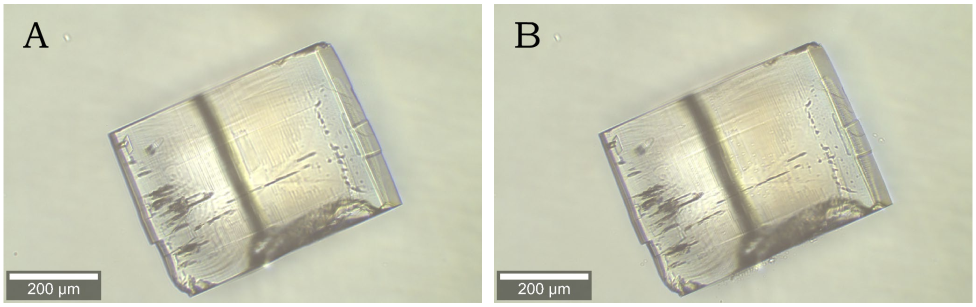

Figure S7: MOF-5 before (A) and after (B) 40 min in DMSO.

Figure S8 shows a crystal before (A) and after (B) being soaked in a DMSO solution of ZnOAc with a molar concentration of 0.6 M. The solution was exposed to air. Significant changes after 40 minutes can be noted.

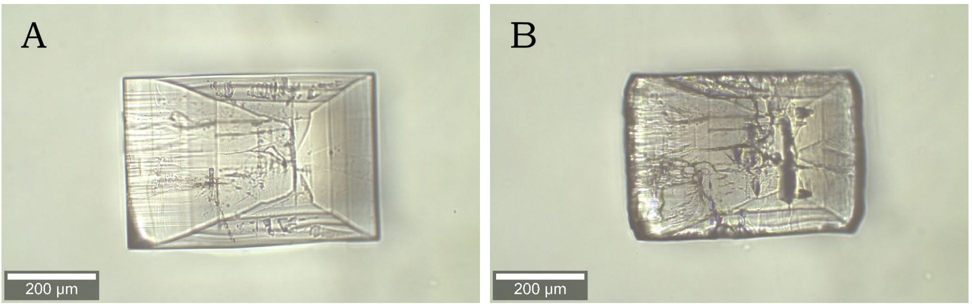

Figure S8: MOF-5 before (A) and after (B) 40 min in a DMSO solution of ZnOAc with a molar concentration of 0.6 M.

Figure S9 shows a crystal before (A) and after (B) being soaked in a DMSO solution of TPA with a molar concentration of 0.2 M. The solution was exposed to air. It can be seen that dissolution occurs largely.

To conclude, the MOF is stable in the mother solution sealed hermetically. DMSO is the best solvent to store the MOF when the solvent is not hermetically sealed.

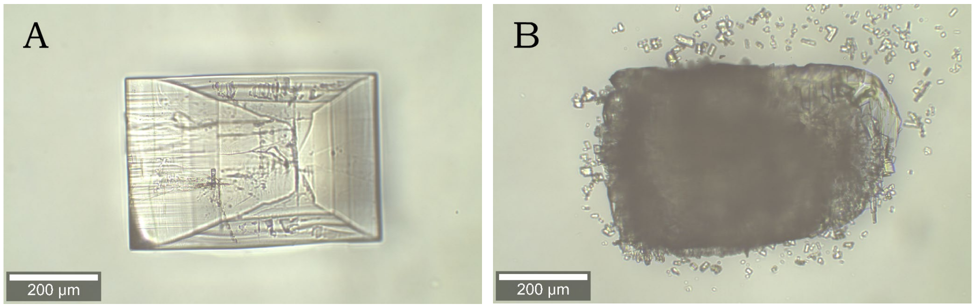

Figure S9: MOF-5 before (A) and after (B) 40 min in a DMSO solution of TPA with a molar concentration of 0.2 M.

Supplementary References

[1] STOE & Cie GmbH. X-area 1.31.175.0, lana 2.6.2.0, 2021.

[2] G. M. Sheldrick. SHELXT — Integrated space-group and crystal-structure determination. *Acta Cryst.*, A71:3–8, 2015.

[3] G. M. Sheldrick. Crystal structure refinement with SHELXL. *Acta Cryst.*, C71:3–8, 2015.

[4] A. L. Spek. Structure validation in chemical crystallography. *Acta Cryst.*, D65:148–155, 2009.

[5] Oleg V. Dolomanov, Luc J. Bourhis, Richard J. Gildea, Judith A. K. Howard, and Horst Puschmann. *OLEX2*: a complete structure solution, refinement and analysis program. *Journal of Applied Crystallography*, 42(2):339–341, Apr 2009.
