# Supplementary material for: Host-guest charge transfer for scalable single crystal epitaxy of a metal-organic framework
Source: Commun Mater. 2024 Oct 9;5(1):220. doi: 10.1038/s43246-024-00657-3 (PMC11488492; doi:10.1038/s43246-024-00657-3)
Supplement: Supplementary file 3 — Description of Additional Supplementary Files [file 43246_2024_657_MOESM3_ESM.pdf]

Description of Additional Supplementary Files

File name: Supplementary Data 1.txt

Description: Crystallographic information file
